# Effects of amikacin, polymyxin-B, and sulbactam combination on the pharmacodynamic indices of mutant selection against multi-drug resistant *Acinetobacter baumannii*

**DOI:** 10.3389/fmicb.2022.1013939

**Published:** 2022-10-20

**Authors:** Shixing Zhu, Chu Song, Jiayuan Zhang, Shuo Diao, Tobias M. Heinrichs, Frederico S. Martins, Zhihua Lv, Yuanqi Zhu, Mingming Yu, Sherwin K. B. Sy

**Affiliations:** ^1^School of Medicine and Pharmacy, Ocean University of China, Qingdao, China; ^2^Department of Pharmaceutics, College of Pharmacy, University of Florida, Gainesville, FL, United States; ^3^Faculty of Pharmaceutical Sciences of Ribeirão Preto, University of São Paulo, São Paulo, Brazil; ^4^Laboratory for Marine Drugs and Bioproducts of Qingdao National Laboratory for Marine Science and Technology, Qingdao, China; ^5^Department of Laboratory Medicine, The Affiliated Hospital of Qingdao University, Qingdao, China; ^6^Department of Statistics, State University of Maringá, Paraná, Brazil

**Keywords:** *Acinetobacter baumannii*, OXA-23, polymyxin-B, amikacin, sulbactam, pharmacodynamics

## Abstract

Amikacin and polymyxins as monotherapies are ineffective against multidrug-resistant *Acinetobacter baumannii* at the clinical dose. When polymyxins, aminoglycosides, and sulbactam are co-administered, the combinations exhibit *in vitro* synergistic activities. The minimum inhibitory concentration (MIC) and mutant prevention concentration (MPC) were determined in 11 and 5 clinical resistant isolates of *A. baumannii* harboring OXA-23, respectively, in order to derive the fraction of time over the 24-h wherein the free drug concentration was within the mutant selection window (*f*T_MSW_) and the fraction of time that the free drug concentration was above the MPC (*f*T_>MPC_) from simulated pharmacokinetic profiles. The combination of these three antibiotics can confer susceptibility in multi-drug resistant *A. baumannii* and reduce the opportunity for bacteria to develop further resistance. Clinical intravenous dosing regimens of amikacin, polymyxin-B, and sulbactam were predicted to optimize *f*T_MSW_ and *f*T_>MPC_ from drug exposures in the blood. Mean *f*T_>MPC_ were ≥ 60% and ≥ 80% for amikacin and polymyxin-B, whereas mean *f*T_MSW_ was reduced to <30% and <15%, respectively, in the triple antibiotic combination. Due to the low free drug concentration of amikacin and polymyxin-B simulated in the epithelial lining fluid, the two predicted pharmacodynamic parameters in the lung after intravenous administration were not optimal even in the combination therapy setting.

## Introduction

*Acinetobacter baumannii* is an opportunistic and dangerous pathogen, causing nosocomial infections, such as meningitis, pneumonia, wound infection, and urinary tract infection; hospital-acquired pneumonia and ventilator-associated pneumonia (HAP/VAP) are the leading cause of death in patients infected with this pathogen and also the leading cause of death in ICU patients (Maragakis and Perl, 2008; Antunes et al., 2014). The overuse of antimicrobial agents has been the primary cause of the emergence of multidrug-resistant (MDR) bacteria (Shi et al., 2020). Antibiotic resistance in *A. baumannii* is mainly due to the production of oxacillinases (OXAs), wherein OXA-23 is the most prevalent worldwide resulting in resistance to carbapenems (Yang et al., 2019).

In addition to OXA-23, carbapenem resistant *A. baumannii* (CRAB) has several resistance mechanisms, including the presence of other β-lactamases (e.g., class B metallo-β-lactamases—MLB, OXA-51-like and OXA-58-like), loss of outer membrane porins, overexpression of efflux pumps and changes in their penicillin-binding proteins (Nguyen and Joshi, 2021). Resistance to colistin and polymyxins is due to complete loss of lipopolysaccharide production or lipid A modification (Moffatt et al., 2010; Qureshi et al., 2015). This rapid adaptive resistance (heteroresistance) of *A. baumannii* to polymyxins is transient and tends to be difficult to detect using standard susceptibility testing methods (Yau et al., 2009; Barin et al., 2013). Consequently, CRAB tends to be also resistant to aminoglycosides, polymyxins, carbapenems, and sulbactam (Penwell et al., 2015; Sun et al., 2020; Fedrigo et al., 2021). Due to the high level of resistance, polymyxin-based antimicrobial combination therapies are the current treatment options against infections due to these pathogens (Cheah et al., 2016; Isler et al., 2019; Menegucci et al., 2019), in order to capitalize on the synergistic activities of combination therapy.

The range of drug concentration between MIC and MPC is defined as the mutant selection window (MSW), wherein selective enrichment and amplification of mutant subpopulations occur (Hesje et al., 2007). A theory was postulated that the lesser time at which the bacteria spent in MSW would translate to a lesser opportunity for them to develop resistance (Hesje et al., 2007). When MPC converged to MIC for all antibiotics in the combination, this suggests that there is no further resistance development (Fedrigo et al., 2021; Feng et al., 2021). Two pharmacodynamic (PD) parameters are often used as an inference of the emergence of resistant mutants: (Antunes et al., 2014) the fraction of time over the 24 h wherein the free drug concentration was within the MSW (*f*T_MSW_); and (Maragakis and Perl, 2008) the fraction of time over the same period wherein the free drug concentration exceeds the MPC (*f*T_>MPC_). An effective antimicrobial combination that restricts resistance development will result in a reduction in *f*T_MSW_ and increased *f*T_>MPC_.

Both colistin and polymyxin-B undergo reabsorption through tubular cells and are nephrotoxic; but polymyxin-B has a lower risk of acute kidney injury (Zavascki and Nation, 2017). In order to lessen nephrotoxic liability of aminoglycosides and polymyxins in combination therapy, we paired amikacin with polymyxin-B. Sulbactam has intrinsic activity against *A. baumannii* by disrupting the bacterial cell wall synthesis and thinning the cell wall to allow companion antibiotics to reach their targets (Lin et al., 2014; Penwell et al., 2015). In this study, we investigated whether the simulated clinical dosing regimens of amikacin/polymyxin-B/sulbactam in a combination setting would optimize the two PD parameters associated with the selection of resistant mutants against MDR *A. baumannii* strains.

## Materials and methods

### Bacterial isolates

This study was performed on MDR *A. baumannii* clinical strains which were collected from the affiliated hospital of Qingdao University. Drug-resistant genes including β-lactamase genes were determined using whole-genome sequencing. Briefly, Wizard® Genomic DNA Purification Kit (Promega) was used to extract the genomic DNA of *A. baumannii* isolates according to manufacturer’s protocol; Illumina MiSeq was used for sequencing. SOAPdenovo2 was used to assemble the qualified reads. Glimmer was applied to predict the coding sequences, and the sequences were further compared against all known drug resistance genes using BLAST to obtain the types of resistance-encoding genes in all studied strains (Feng et al., 2021). *E. coli* ATCC 25922 and *A. baumannii* ATCC19606 were selected as quality control strains for antimicrobial susceptibility tests. This study was approved by the Ethics Committee of the Affiliated Hospital of Qingdao University and strictly in accordance with the Helsinki declaration and its appendices.

### Antimicrobial agents

Analytical-grade amikacin, polymyxin-B, and sulbactam were purchased from the Shanghai Macklin Biochemical Co. Ltd. (Shanghai, China). Stock solutions of amikacin, polymyxin B, and sulbactam were prepared separately according to CLSI guidelines (CLSI, 2020).

### Susceptibility testing

MIC determination under the CLSI guidelines (CLSI, 2020) was carried out using a checkerboard method. Susceptibility tests of amikacin, polymyxin-B, and sulbactam alone or as double and triple combinations were conducted in triplicate for each of the *A. baumannii* isolates using a sterile 96-well microdilution plate. The concentration ranges of amikacin and polymyxin-B tested were 1 to 128 and 1 to 64 mg/L, respectively. The test was carried out at a fixed sulbactam concentration of 4 mg/L, when sulbactam was included in the combination. A fixed 4 mg/L sulbactam concentration was selected, as sulbactam clinical dosing recommendation used in this study was previously shown to achieve ≥90% probability of target attainment (PTA) for MIC of 4 mg/L (Yokoyama et al., 2015).

A standard inoculum of 0.5 McFarland was measured using a nephelometer (bioMérieux, Marcy l’Etoile, France); this inoculum was diluted into each well to achieve a final concentration of 5 × 10^5^ cfu/ml. The plate was then incubated at 35°C ± 2°C for 20 h.

The fractional inhibitory concentration index (FICI) was calculated from the results of the checkerboard method, according to the following equation to classify the antimicrobial synergy of the combination:


$$\begin{array}{l} FICI=\frac{MIC\;ofantibiotic1\;incombination}{MIC\;ofantibiotic1\;alone}\\ \;\;\;\;\;\;\;\;\;\;\;\;+\frac{MIC\;ofantibiotic2\;incombination}{MIC\;ofantibiotic2\;alone}\\ \;\;\;\;\;\;\;\;\;\;\;\;+\frac{MIC\;ofantibiotic3\;incombination}{MIC\;ofantibiotic3\;alone}. \end{array}$$

A FIC index of ≤0.5 indicates synergism, >0.5–1 is an additive effect, >1 to <2 refers to indifference, and ≥2 is antagonism.

The MPC of amikacin and polymyxin-B alone and in combination with and without 4 mg/l sulbactam were determined using a final high-density inoculum of ≥10^10^ cfu/ml in a subset of five isolates. The high inoculum size ensured the emergence of the first-step mutants (Dong et al., 1999). About 100 μl of the high-density inoculum was plated onto the Mueller-Hinton agar plates containing antimicrobial concentrations at 1×, 2×, 4×, 8×, 16×, and 32 × MIC. MPC was determined as the lowest antimicrobial concentration that completely prevented bacterial growth after 72 h incubation at 35°C ± 2°C.

### Time-kill kinetics

The *in vitro* dynamic time-course of two *A. baumannii* isolates (A and E) in response to polymyxin-B, amikacin and sulbactam alone and their combination were studied by time-kill kinetics. The experiment consisted of five groups including control, amikacin, polymyxin-B, amikacin plus polymyxin-B combination, and the combination consisting of amikacin, polymyxin-B plus 4 mg/L sulbactam. The concentrations of amikacin and polymyxin-B were tested at their respective MIC and 2 × MIC. The constant concentration time-kill studies were carried out as follows: *A. baumannii* isolates A and E were cultured in Mueller Hinton broth at 35°C ± 2°C for 1 h to achieve logarithmic growth. Before adding the drug, the inoculum was initially standardized to 5 × 10^5^ cfu/ml.

The drug concentrations against isolate A were as follows: 128 mg/L amikacin, 8 mg/L polymyxin-B, 1 mg/L amikacin and 4 mg/L polymyxin-B with and without 4 mg/L sulbactam. For 2 × MIC test, the drug concentrations for amikacin and polymyxin-B were doubled while sulbactam concentration was not changed.

For isolate E, the drug concentrations were 128 mg/L amikacin, 16 mg/l polymyxin-B, 32 mg/L amikacin and 4 mg/L polymyxin-B in combination, and 1 mg/L amikacin and 2 mg/L polymyxin-B with 4 mg/L sulbactam. Both amikacin and polymyxin-B concentrations were doubled for the 2 × MIC cohort without changing sulbactam concentration.

The flask was incubated at 35°C ± 2°C with a constant shaking at 180 rpm. At 0, 2, 4, 6, 8, and 24 h post-drug administration, the bacterial concentrations were determined. 200 μl of samples were taken from the flask at the pre-determined time points and then diluted 10-fold with fresh normal saline in sterile environment. 100 μl of the diluted bacterial solution was spread evenly on Muller-Hinton agar. After incubation at 35°C ± 2°C for 24 h, the colonies on the petri dish were counted. All time-kill experiments were performed in triplicate. The concentration of bacteria at each time point was calculated according to their respective dilution; the final results were reported in cfu/ml.

### Population pharmacokinetic simulations and pharmacodynamic indices

The virtual population consisted of 10,000 virtual patients, assuming a 50:50 male to female ratio. We assumed height distributions of males and females of 176.3 ± 17√4,482 cm (mean ± SD, where SD is computed as SE√*n*) and 162.2 ± 0.16√4,857 cm, respectively (McDowell et al., 2008). Body weight (WT) was determined from their height (HT) using the following equations: $WT_{male}=\exp\left(3.28+1.92\log HT_{male}\right)$ ; and $WT_{female}=\exp\left(3.49+1.45\log HT_{female}\right)$ , for male and female, respectively (Diverse Populations Collaborative Group, 2005). Inter-individual variability in body weight was simulated by $WT_{i}=WT\exp\left(\eta_{i}\right)$ , wherein η is normally distributed with 0 mean and SD of 0.14 and 0.17, for male and female, respectively (Sy et al., 2014). Creatinine clearance (CL_CR_) was simulated using a uniform distribution ranging from 30 to 150 ml/min. The time-course of drug concentrations over 6 days was simulated using reported population pharmacokinetic (PK) models with WT and CL_CR_ as covariates of the model parameters. Simulation over 6 days ensured that steady-state was achieved. Amikacin is not recommended for patients whose CL_CR_ is below 30 ml/min. A brief description of population pharmacokinetic models is available in the Supplementary Material (SM). The intravenous dosing regimens for amikacin and sulbactam by renal function as well as two dosing regimens of polymyxin-B are listed in Table 1. The time course of drug concentration for 10,000 virtual individuals per dosing regimen was simulated over a day.

**Table 1:** Dosing regimens of amikacin/polymyxin-B/sulbactam used in simulation by creatinine clearance category.

Creatinine clearance	Dosing regimens
**Amikacin/sulbactam**	
≥60 ml/min	15 mg/kg q24 h/3 g q8h as continuous infusion
40 to 59 ml/min	15 mg/kg q36 h/3 g q8h as 3 h infusion
30 to 39 ml/min	15 mg/kg q48 h/3.5 g q12h as 4 h infusion
**Polymyxin-B**	
All renal function	Loading dose 2.5 mg/kg followed by 1.5 mg/kg q12 h at 12 h as 1 h infusion
All renal function	Loading dose 2.0 mg/kg followed by 1.25 mg/kg q12 h at 12 h as 1 h infusion

Hospital-acquired and ventilator-acquired pneumonia are often caused by *A. baumannii* infecting the lungs. Epithelial lining fluid (ELF) is considered an important site of common extracellular infection (Rodvold et al., 2011; Sarshar et al., 2021). Pharmacodynamic evaluation should also consider drug exposure in the ELF, in addition to drug exposure in the blood. Free drug concentrations in the plasma and ELF were used to compute the pharmacodynamic parameters. ELF to plasma penetration used for polymyxin-B, sulbactam and amikacin were 60%, 52%, and 18%, respectively (He et al., 2013; Rodvold et al., 2018; Najmeddin et al., 2020). Plasma protein binding of sulbactam was 32%, whereas amikacin protein binding was negligible. Polymyxin-B plasma protein binding is highly variable, ranging from 50% to 92% (Zavascki et al., 2008; Sandri et al., 2013; Abodakpi et al., 2015). The simulation of polymyxin-B in the ELF assumed that unbound polymyxin-B in the presence of mucin was 15% (Huang et al., 2015; Samad et al., 2019). The high mucin binding of polymyxin-B is a conservative estimate of free polymyxin-B concentration in the ELF.

The target PD indices of polymyxin-B, amikacin, and sulbactam were ≥8.2 *f*AUC/MIC, ≥8 *f*C_max_/MIC and 60% *f*T_>MIC_, respectively (Bergen et al., 2012; Yokoyama et al., 2014, 2015; Kato et al., 2017). We assumed a fixed MIC of 4 mg/L for sulbactam, corresponding to the fixed 4 mg/L tested in the *in vitro* susceptibility determination. These values were used in the determination of probability of target attainment (PTA).

### Pharmacodynamic parameters for suppression of emergence of resistant mutant

For the suppression of emergence of resistant mutants, the two PD parameters *f*T_MSW_ and *f*T_>MPC_ were determined. *f*T_MSW_ was computed as the difference between *f*T_>MPC_ and *f*T_>MIC_, only if *f*T_>MPC_ was >0% for all virtual population. The summary statistics of the PD parameters for each of the selected isolates were reported based on simulations of 10,000 concentration-time profiles for each dosing regimen. Because polymyxin-B protein binding was highly variable, a sensitivity analysis was carried out to evaluate the effect of protein binding on the two PD parameters.

### Software

The pharmacokinetic simulations and pharmacodynamic analyses were carried out using the RxODE package and user-defined functions in R (4.1.2).

## Results

### *In vitro* antimicrobial susceptibility

All of the 11 *A. baumannii* isolates showed significant drug resistance to amikacin and polymyxin B (Table 2). Drug resistance genes are summarized in the Supplementary Table S2. The control *E. coli* (ATCC25922) and *A. baumanni* (ATCC19606) strains were susceptible to all of the above antimicrobial agents. The MIC of amikacin alone in most of the clinical isolates was greater than 128 mg/L; the MIC of polymyxin B alone in most isolates ranged from 2 to >16 mg/L. There were no changes in amikacin MIC with the addition of sulbactam which was fixed at 4 mg/L, whereas the addition of sulbactam to polymyxin-B slightly reduced the MIC of polymyxin-B in some strains. The combination of amikacin and polymyxin-B reduced the MIC to the breakpoints of either amikacin (16 mg/L) or polymyxin B (2 mg/L) in 8 and 3 of the 11 strains, respectively. The addition of sulbactam to the combination of amikacin and polymyxin B further lowered the MIC of amikacin and polymyxin B to the clinical breakpoints of amikacin and polymyxin B in 10 of 11 strains and 5 of 11 strains, respectively.

**Table 2:** Minimum inhibitory concentrations of amikacin and polymyxin B alone or in combination with or without sulbactam (fixed at 4 mg/l) against carbapenem-resistant *Acinetobacter baumannii* isolates and fractional inhibitory concentration index (wherein sulbactam was fixed at 4 mg/L).

Strains	Amikacin					Polymyxin B					Sulbactam	Synergism analysis	
	MIC (mg/L)					MIC (mg/L)					MIC (mg/L)		
	Amikacin alone	Amikacin plus sulbactam	Amikacin plus polymyxin-B	Amikacin/polymyxin-B/sulbactam	Fold reduction in amikacin MIC between triple combination and amikacin alone	Polymyxin-B alone	Polymyxin-B plus sulbactam	Polymyxin-B plus amikacin	Polymyxin-B/ amikacin/ sulbactam	Fold reduction in polymyxin B MIC between triple combination and polymyxin-B alone	Sulbactam alone	FICI^‡^	S or I based on FICI
*E. coli* ATCC25922	≤1	-	-	-	-	≤1	-	-	-	-	32	-	-
*Acinetobacter baumannii*													
*ATCC19606*	4	-	-	-	-	2	-	-	-	-	64	-	-
A	>128	>128	1	1	128	8	8	4	4	2	>64	0.5703	A
C	>128	>128	64	64	2	8	8	8	8	1	>64	1.5625	I
E	>128	>128	32	1	128	16	4	2	2	8	>64	0.1953	S
F	>128	>128	128	4	32	8	8	8	4	2	>64	0.5938	A
G	>128	>128	8	8	16	4	4	2	2	2	>64	0.6250	A
2	16	16	2	1	16	16	8	4	2	8	>64	0.2500	S
12	>128	>128	4	2	64	16	8	2	2	8	>64	0.2031	S
13	>128	>128	4	1	128	16	8	8	4	4	>64	0.3203	S
20	>128	>128	2	1	128	8	8	4	1	8	>64	0.1953	S
21	>128	>128	1	1	128	16	8	8	4	4	>64	0.3203	S
22	>128	>128	2	1	128	8	8	8	4	2	>64	0.5703	A
MIC50	>128	>128	4	1		8	8	4	4		>64		
MIC90	>128	>128	64	8		16	8	8	4		>64		

MIC, minimum inhibitory concentration; FICI, fractional inhibitory concentration index; A, additive effects; S, synergy; I, indifferent. CLSI and EUCAST breakpoints for interpretation of polymyxin-B MIC results: ≤2 mg/L (intermediate), >2 mg/L (resistant); and amikacin MIC results: ≤8 mg/L (susceptible), and <8 mg/L (resistant) for *A. baumannii*.

‡ FICI score was computed using the reduced MICs of amikacin, polymyxin-B and sulbactam in the triple combination relative to amikacin, polymyxin-B, and sulbactam alone.

The MPC values of amikacin, polymyxin B alone or in combination with sulbactam (fixed at 4 mg/L) in 5 clinical isolates are shown in Table 3. The MPC values of amikacin alone were all higher than 128 mg/L, whereas that of polymyxin B ranged from 16 to 64 mg/L. The combination of amikacin/polymyxin B with sulbactam significantly reduced the MPC values of all antimicrobial agents to 2 to 4 mg/L. The MPCs in co-administration were much lower than those in monotherapy. There were remarkable reductions in both MIC and MPC values in the triple combination.

**Table 3:** Mutant prevention concentrations of amikacin and polymyxin B alone or in combination with or without sulbactam (fixed at 4 mg/L) against five *Acinetobacter baumannii* isolates harboring OXA-23 and other serine-β-lactamases.

Strains	Amikacin				Polymyxin-B			
	MPC (mg/L)/MIC (mg/L)^‡^				MPC (mg/L)/MIC (mg/L)^‡^			
	Amikacin alone	Amikacin/polymyxin-B	Amikacin/polymyxin-B/sulbactam	Fold reduction in amikacin MPC	Polymyxin-B alone	Polymyxin-B/ amikacin	Polymyxin-B/ amikacin/ sulbactam	Fold reduction in polymyxin-B MPC
A	>128/>128	8/1	4/1	>32	32/8	8/4	4/4	8
E	>128/>128	64/32	4/1	>32	64/16	8/4	4/2	16
2	>128/16	4/2	4/1	>32	64/16	4/4	4/2	16
12	>128/>128	4/4	2/2	>64	32/16	4/2	2/2	16
20	>128/>128	4/2	4/1	>32	16/8	8/4	2/1	8

MPC, mutant prevention concentration; MIC, Minimum inhibitory concentration.

‡ Values reported as MPC (mg/L)/MIC (mg/L); these values do not refer to ratio of the two.

### Time-kill kinetics

**Figure 1:**
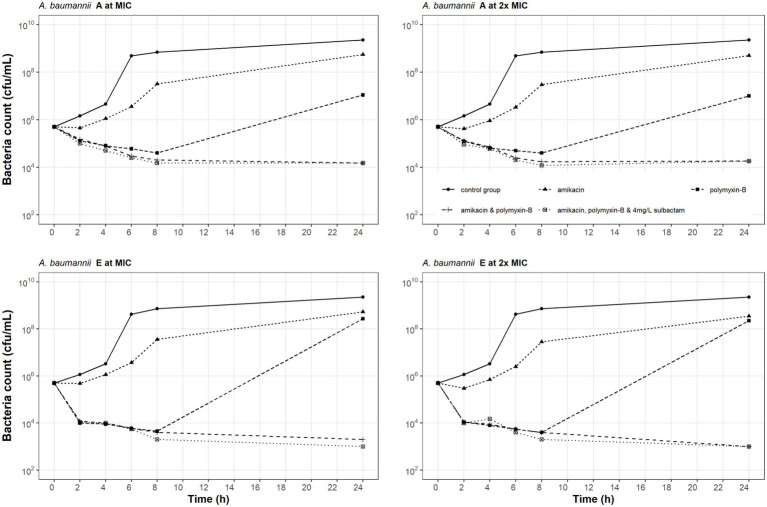
Static-concentration time-kill kinetics of amikacin and polymyxin-B alone and in combination at their respective MIC and 2 × MIC and also as triple combination with 4 mg/L sulbactam against two *Acinetobacter baumannii* isolates. Monotherapy MICs for amikacin and polymyxin-B were >128 and 8 mg/L for isolate A, and >128 and 16 mg/L for isolate E; MICs in the double combination were 1 and 4 mg/L for isolate A and 32 and 4 mg/L for isolate E; MICs in the triple combination were 1 and 4 mg/L for isolate A and 1 and 2 mg/L for isolate E, respectively.

Time-kill experiments evaluated the effects of amikacin, polymyxin-B alone and their combination with or without sulbactam (fixed at 4 mg/L) at their respective MIC and 2 × MIC (Table 2) on the bacterial dynamics of two MDR *A. baumannii* isolates (A and E). The results of the time-kill kinetics are shown in Figure 1. For bacteria treated with amikacin alone, no restraint on their growth at 128 and 256 mg/L amikacin concentration was observed. After polymyxin-B administration alone (MIC: 32 and 64 mg/L), the growth of isolates A and E were significantly suppressed before 8 h, but bacteria regrew to a density > 10^7^ cfu/ml at 24 h. These results suggest that there may be hetero-resistance to polymyxin-B in the two *A. baumannii* isolates.

The combination of amikacin and polymyxin-B (1 and 4 mg/L, respectively for isolate A; 1 and 2 mg/L, respectively for isolate E) with or without 4 mg/L sulbactam inhibited bacterial growth. For isolate A, the addition of sulbactam did not enhance the bactericidal effect of amikacin and polymyxin-B combination. At 2 × MIC for amikacin and polymyxin-B (2 and 8 mg/L, respectively for isolate A; 2 and 4 mg/L, respectively for isolate E), bactericidal activity was not improved compared to the results at MIC. The addition of sulbactam to amikacin and polymyxin-B combination enhanced the bactericidal effect against isolate E.

### Pharmacodynamic analysis of resistant mutant selection

**Figure 2:**
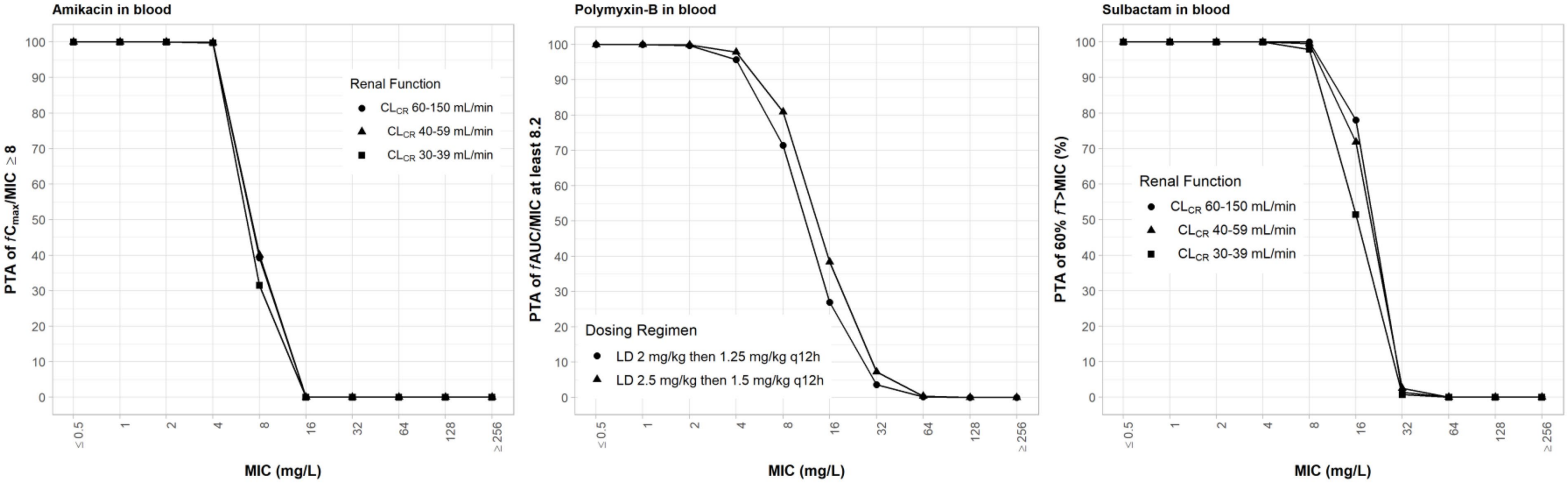
Probability of target attainment (PTA) of 60% *f*T_>MIC_ and 8 *f*C_max_/MIC for sulbactam and amikacin dosing regimens by renal function category, respectively, and PTA of *f*AUC/MIC of at least 8.2 for polymyxin-B dosing regimens. PTA values were computed based on steady-state drug concentrations in the blood. LD, loading dose; CL_CR_, creatinine clearance.

With the exception of polymyxin-B, all other antibiotics are dosed according to renal function (Table 1). Since polymyxin-B has high tubular reabsorption and is eliminated *via* non-renal pathways, we assessed the higher and lower ranges of the dosing regimens applying to all renal categories associated with amikacin dosing regimens (Tsuji et al., 2019). The high-dose regimens of sulbactam were selected based on achieving a PTA ≥ 90% for 60% *f*T_>MIC,_ assuming an MIC fixed at 4 mg/L (Yokoyama et al., 2015) in both plasma and ELF. Amikacin dosing regimens were selected based on the recommended regimens that achieve sufficient coverage. The dosing regimens of amikacin and sulbactam were simulated according to the three renal function categories; 100% PTA was achieved for MIC of 4 and 8 mg/L, respectively (Figure 2). The probability for steady-state trough amikacin concentrations greater than or equal to 10 mg/L which is associated with amikacin toxicity, is less than 10% (Supplementary Figure S1). For polymyxin-B exposures in the blood, ≥90% PTA was achieved at 4 mg/L, for the dosing regimens listed in Table 1. The dosing regimens of amikacin, polymyxin-B, and sulbactam are expected to provide sufficient PTA in the combination therapy against the five isolates, except for isolate E in the double combination wherein amikacin MIC is 32 mg/L (Table 3).

When the simulated drug concentrations were lower than the MPC, *f*T_>MPC_ and *f*T_MSW_ were 0% and not determinable, respectively. *f*T_MSW_ is not determinable in this situation because it can result in an artificially small value. In the combination therapy scenario, these two PD indices were determinable. The PD parameters in monotherapy were not included in Tables 4, 5 due to their high MPC. Table 4 lists the PD parameters (*f*T_MSW_ and *f*T_>MPC_) for amikacin in double and triple combinations in plasma for the dosing regimens associated with the renal function groups. In all isolates, mean *f*T_>MPC_ values were over 64% after sulbactam added. In isolate 12, the *f*T_MSW_ was 0%, whereas mean *f*T_MSW_ values were <27% for the other isolates. With the addition of sulbactam, these PD parameters are optimized by increasing *f*T_>MPC_ while decreasing *f*T_MSW_.

**Table 4:** Pharmacodynamic parameters *f*T_MSW_ and *f*T_>MPC_ based on MIC and MPC of amikacin^‡^ in plasma against five *Acinetobacter baumannii* isolates harboring OXA-23 and other serine-β-lactamases.

	Double-combination of amikacin with polymyxin-B		Triple-combination of amikacin with polymyxin-B and sulbactam	
Bacteria isolate	*f*T_MSW_	*f*T_>MPC_	*f*T_MSW_	*f*T_>MPC_
**CL**_**CR**_ **≥ 60 to 150 ml/min**				
A	49.88 ± 18.3%	41.02 ± 22.17%	26.85 ± 16.37%	64.05 ± 24.06%
E	ND	5.0 ± 4.11%	26.85 ± 16.37%	64.05 ± 24.06%
2,20	16.88 ± 9.89%	64.05 ± 24.06%	26.85 ± 16.37%	64.05 ± 24.06%
12	0%	64.05 ± 24.06%	0%	80.93 ± 20.4%
**CL**_**CR**_**40 to 59 ml/min**				
A	45.92 ± 18.38%	48.12 ± 21.97%	22.77 ± 15.92%	71.27 ± 22.18%
E	ND	4.94 ± 4.7%	22.77 ± 15.92%	71.27 ± 22.18%
2,20	15.03 ± 10.0%	71.27 ± 22.18%	22.77 ± 15.92%	71.27 ± 22.18%
12	0%	71.27 ± 22.18%	0%	86.31 ± 17.46%
**CL**_**CR**_**30 to 39 ml/min**				
A	46.66 ± 17.2%	46.43 ± 20.79%	23.76 ± 15.27%	69.33 ± 21.95%
E	ND	4.3 ± 4.27%	23.76 ± 15.27%	69.33 ± 21.95%
2,20	15.46 ± 9.43%	69.33 ± 21.95%	23.76 ± 15.27%	69.33 ± 21.95%
12	0%	69.33 ± 21.95%	0%	84.79 ± 18.11%

CL_CR_, creatinine clearance; MIC, minimum inhibitory concentration; MPC, mutant prevention concentration; *f*T_MSW_, fraction of time within 24-h that the drug concentration is within mutant selection window; *f*T_>MPC_, fraction of time within 24-h that the drug concentration is above MPC; ND, not determinable.

‡ See Table 1 for list of dosing regimens by renal function category; simulations were based on assumption of 90% PTA achieved in the plasma using polymyxin-B dosing regimens: loading dose 2.5 mg/kg followed by 1.5 mg/kg q12h at 12 h as 1 h infusion.

**Table 5:** Pharmacodynamic parameters *f*T_MSW_ and *f*T_>MPC_ based on MIC and MPC of polymyxin-B^‡^ in plasma against five *Acinetobacter baumannii* isolates harboring OXA-23 and other serine-β-lactamases.

	Double-combination of polymyxin-B with amikacin		Triple-combination of polymyxin-B with amikacin and sulbactam		
Bacteria isolate	*f*T_MSW_	*f*T_>MPC_	*f*T_MSW_	*f*T_>MPC_	
**Loading dose 2.5 mg/kg followed by 1.5 mg/kg q12h at 12 h as 1 h infusion**					
A	28.44 ± 28.28%	61.54 ± 36.02%		0%	89.98 ± 22.35%
E	28.44 ± 28.28%	61.54 ± 36.02%		8.81 ± 19.69%	89.98 ± 22.35%
2	0%	89.98 ± 22.35%		8.81 ± 19.69%	89.98 ± 22.35%
12	8.81 ± 19.69%	89.98 ± 22.35%		0%	98.79 ± 7.31%
20	28.44 ± 28.28%	61.54 ± 36.02%		1.11 ± 7.02%	98.79 ± 7.31%
**Loading dose 2 mg/kg followed by 1.25 mg/kg q12h at 12 h as 1 h infusion**					
A	33.7 ± 27.85%	49.9 ± 36.12%		0%	83.6 ± 27.73%
E	33.7 ± 27.85%	49.9 ± 36.12%		13.9 ± 23.57%	83.6 ± 27.73%
2	0%	83.6 ± 27.73%		13.9 ± 23.57%	83.6 ± 27.73%
12	13.9 ± 23.57%	83.6 ± 27.73%		0%	97.5 ± 10.94%
20	33.7 ± 27.85%	49.9 ± 36.12%		2.29 ± 10.2%	97.5 ± 10.94%

MIC, minimum inhibitory concentration; MPC, mutant prevention concentration; *f*T_MSW_, fraction of time within 24 h that the drug concentration is within mutant selection window; *f*T_>MPC_, fraction of time within 24-h that the drug concentration is above MPC; ND, not determinable.

‡ See Table 1 for list of dosing regimens by renal function category; simulations were based on assumption of 90% PTA achieved in the plasma using amikacin/sulbactam dosing regimens of 20 mg/kg/day q24h/3 g q8 h as 3 h infusion in CL_CR_ > 50 to 150 ml/min.

The PD parameters of polymyxin-B against these 5 isolates were calculated on the assumption that the plasma protein binding of polymyxin-B is 60% (Table 5). The MSW was closed in 1/5 isolates for polymyxin-B and amikacin together, and the MSW was significantly reduced in 4/5 isolates when the triple combination was used. For majority of the isolates, the mean *f*T_>MPC_ were >89% and >83% for the polymyxin-B dosing regimen consisting of loading dose 2.5 mg/kg followed by 1.5 mg/kg q12h at 12 h and loading dose 2 mg/kg followed by 1.25 mg/kg q12h at 12 h, respectively. For isolate 12 and 20, mean *f*T_>MPC_ was >95% due to very low MPC in the triple combination.

**Figure 3:**
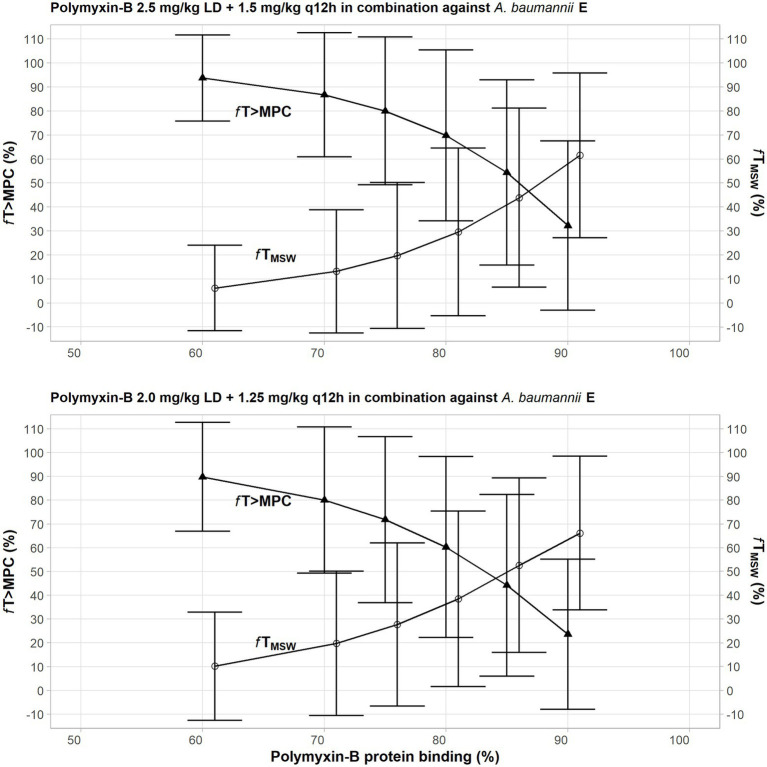
Sensitivity analysis to evaluate effect of variability in polymyxin plasma protein binding on the pharmacodynamic parameters *f*T_MSW_ and *f*T_>MPC_ after polymyxin dosing regimens in combination therapy consisting of loading dose 2.5 mg/kg followed by 1.5 mg/kg q12h at 12 h (top) and loading dose 2 mg/kg followed by 1.25 mg/kg q12h at 12 h (bottom) against *Acinetobacter baumannii* 20. The models assumed polymyxin-B MIC of 1 mg/L and MPC of 4 mg/L, whereas amikacin MIC and MPC were both 4 mg/L with or without 4 mg/L sulbactam. In this scenario, the proposed dosing regimens of both amikacin and sulbactam can achieve PTA ≥ 90%.

Given that protein binding of polymyxin-B is highly variable, a sensitivity analysis was performed to illustrate the effects of variance of protein binding on these two PD parameters, as shown in Figure 3. We selected isolate E, since the MSW was not closed in the amikacin/polymyxin-B combination with and without sulbactam. When polymyxin-B plasma protein binding increased from 60% to 90%, the *f*T_>MPC_ of polymyxin-B combined with amikacin decreased from over 90% to slightly over 30%, whereas *f*T_MSW_ increased from <10 to >60% for the 2.5 mg/kg loading dose followed by 1.5 mg/kg q12h dosing regimen. For the 2.0 mg/kg loading dose followed by 1.25 mg/kg q12h regimen, the *f*T_>MPC_ of polymyxin-B combined with amikacin and sulbactam decreased from 90% to <30%, and *f*T_MSW_ increased from 10% to >60%. The results indicated a sensitivity of the two PD parameters to the availability of free drug concentration of polymyxin-B in the blood.

### Pharmacokinetic and pharmacodynamic analyses of drugs in epithelial lining fluid

**Figure 4:**
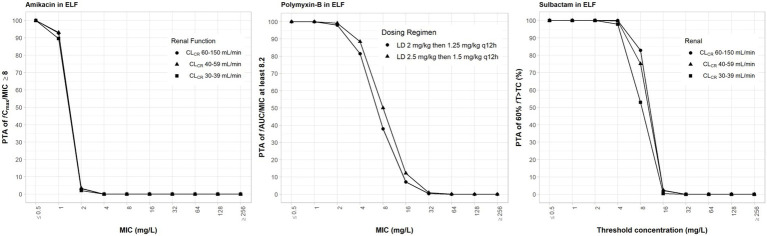
Probability of target attainment (PTA) of 60% *f*T_>MIC_ and 8 *f*C_max_/MIC for sulbactam and amikacin dosing regimens by renal function category, respectively, and PTA of *f*AUC/MIC of at least 8.2 for polymyxin-B dosing regimens. PTA values were computed based on steady-state drug concentrations in the epithelial lining fluid and their respective epithelial lining fluid penetration. LD, loading dose; CL_CR_, creatinine clearance.

Amikacin has a low ELF penetration and is highly bound to mucin. Consequently, free amikacin in the lung is very low. A ≥ 95% PTA can only be achieved at MIC ≤1 mg/L in the ELF (Figure 4). The PD parameters of amikacin in the ELF are not optimal. Mean *f*T_>MPC_ were <15% (Table 6). Because many simulated individual C_max_ were below MPC, *f*T_MSW_ values were not determinable.

**Table 6:** Pharmacodynamic parameters *f*T_MSW_ and *f*T_>MPC_ based on MIC and MPC of amikacin in epithelial lining fluid against five *Acinetobacter baumannii* isolates harboring OXA-23 and other serine-β-lactamases.

	Double-combination of amikacin with polymyxin-B		Triple-combination of amikacin with polymyxin-B and sulbactam	
Bacteria isolate	*f*T_MSW_	*f*T_>MPC_	*f*T_MSW_	*f*T_>MPC_
**CL**_**CR**_**60 to 150 ml/min**				
A	ND	2.21 ± 1.62%	ND	10.35 ± 8.05%
E	ND	0%	ND	10.35 ± 8.05%
2,20	ND	10.35 ± 8.05%	ND	10.35 ± 8.05%
12	0%	10.35 ± 8.05%	0%	29.17 ± 18.34%
**CL**_**CR**_**40 to 59 ml/min**				
A	ND	1.81 ± 1.73%	ND	11.71 ± 9.73%
E	ND	0%	ND	11.71 ± 9.73%
2,20	ND	11.71 ± 9.73%	ND	11.71 ± 9.73%
12	0%	11.71 ± 9.73%	0%	37.74 ± 19.1%
**CL**_**CR**_**30 to 39 ml/min**				
A	ND	1.49 ± 1.56%	ND	11.09 ± 9.32%
E	ND	0%	ND	11.09 ± 9.32%
2,20	ND	11.09 ± 9.32%	ND	11.09 ± 9.32%
12	0%	11.09 ± 9.32%	0%	33.81 ± 17.92%

CL_CR_, creatinine clearance; MIC, minimum inhibitory concentration; MPC, mutant prevention concentration; *f*T_MSW_, fraction of time within 24 h that the drug concentration is within mutant selection window; *f*T_>MPC_, fraction of time within 24-h that the drug concentration is above MPC; ND, not determinable.

‡ See Table 1 for list of dosing regimens by renal function category; simulations were based on assumption of 90% PTA achieved in the plasma using polymyxin-B dosing regimens: loading dose 2.5 mg/kg followed by 1.5 mg/kg q12h at 12 h as 1 h infusion.

The PD parameters of polymyxin-B were also not optimal (Table 7) due to low ELF penetration and high mucin binding. The addition of sulbactam significantly increased *f*T_>MPC_ in isolates A, E, 12, and 20 for the two dosing regimens. Sulbactam is not expected to improve these PD parameters because free drug concentration in the ELF is very low for both amikacin and polymyxin-B.

**Table 7:** Pharmacodynamic parameters *f*T_MSW_ and *f*T_>MPC_ based on MIC and MPC of polymyxin-B in epithelial lining fluid against five *Acinetobacter baumannii* isolates harboring OXA-23 and other serine-β-lactamases.

	Double-combination of polymyxin-B with amikacin		Triple-combination of polymyxin-B with amikacin and sulbactam		
Bacteria isolate	*f*T_MSW_	*f*T_>MPC_	*f*T_MSW_	*f*T_>MPC_	
**Loading dose 2.5 mg/kg followed by 1.5 mg/kg q12h at 12 h as 1 h infusion**					
A	ND	3.13 ± 8.18%		0%	20.86 ± 25.67%
E	ND	3.13 ± 8.18%		35.12 ± 26.07%	20.86 ± 25.67%
2	0%	20.86 ± 25.67%		35.12 ± 26.07%	20.86 ± 25.67%
12	35.12 ± 26.07%	20.86 ± 25.67%		0%	56.0 ± 36.68%
20	ND	3.13 ± 8.18%		30.58 ± 28.25%	56.0 ± 36.68%
**Loading dose 2 mg/kg followed by 1.25 mg/kg q12h at 12 h as 1 h infusion**					
A	ND	1.19 ± 4.63%		0%	13.43 ± 19.94%
E	ND	1.19 ± 4.63%		ND	13.43 ± 19.94%
2	0%	13.43 ± 19.94%		ND	13.43 ± 19.94%
12	ND	13.43 ± 19.94%		0%	44.37 ± 35.39%
20	ND	1.19 ± 4.63%		34.76 ± 27.25%	44.37 ± 35.39%

MIC, minimum inhibitory concentration; MPC, mutant prevention concentration; *f*T_MSW_, fraction of time within 24 h that the drug concentration is within mutant selection window; *f*T_>MPC_, fraction of time within 24-h that the drug concentration is above MPC; ND, not determinable.

‡ *f*T_MSW_ in some simulated individual profiles were 0 due to 0 *f*T_>MPC_.

‡ See Table 1 for list of dosing regimens by renal function category; simulations were based on assumption of 90% PTA achieved in the plasma using amikacin/sulbactam dosing regimens of 20 mg/kg/day q24h/3 g q8h as 3 h infusion in CL_CR_ > 50 to 150 ml/min.

## Discussion

In recent years, MDR *A. baumannii* especially those harboring OXA-23 carbapenemase increasingly contributed to serious nosocomial and community-acquired infections (Peleg et al., 2008; Howard et al., 2012; Al Atrouni et al., 2016; Ning et al., 2017; Yang et al., 2019; Palmieri et al., 2020). Consequently, combination antimicrobial therapy is more frequently used in the clinic to treat infections due to MDR *A. baumannii* (Penwell et al., 2015; Cheah et al., 2016; Srinivas and Rivard, 2017; Caballero et al., 2018; Sun et al., 2020; Wang et al., 2020; Fedrigo et al., 2021).

Sulbactam has intrinsic activity against *A. baumannii* (Lin et al., 2014; Penwell et al., 2015) but OXA-23, TEM-1 and ADC can confer sulbactam resistance in *A. baumannii* (Yang et al., 2019). The overproduction of cross-linked peptidoglycan keeps antibiotics on the cell surface and prevents adequate antibiotics from entering the cell to reach their critical target. For example, amikacin targets the bacterial ribosomal function center to inhibit protein synthesis (Shakil et al., 2008; Prokhorova et al., 2017) and its target requires drug entry into the bacterial cell. The deceased cell wall thickening is functionally relevant in conferring susceptibility to polymyxins and aminoglycosides. Previous metabonomic study showed that the synergistic effects of polymyxin-aminoglycoside combination were primarily due to disruption cell membrane biogenesis followed by imbalances of central carbohydrate metabolism, amino sugar and nucleotide metabolic pathways (Hussein et al., 2019).

Polymyxins are being reconsidered as antibiotics of last resort in cases where multidrug-resistant infections are untreatable with other antibiotics (Poirel et al., 2017). However, polymyxin monotherapy often resulted in transient emergence of hetero-resistance. CLSI (2020) removed susceptible category for polymyxin due to the number of treatment failures and the development of resistance resulting from polymyxin monotherapy (Satlin et al., 2020). Bacterial heteroresistance to polymyxins is commonly believed to be due to the modification of bacterial outer membrane lipopolysaccharides (Srinivas and Rivard, 2017), by inducing lipid A diacylation to impact drug penetration and to generate high level resistance to polymyxins (Olaitan et al., 2014; Han et al., 2017). Heteroresistance is difficult to be detected using standard susceptibility testing methods. A follow-up study investigates the effects of the same antibiotic combination on the time-dependent changes in metabolomic profiles of *A. baumannii* isolates (unpublished data).

By combining colistin and amikacin, Chung and Ko showed that the combination can effectively eradicate *A. baumannii* persister cells and restrict heteroresistance emergence (Chung and Ko, 2019). Another group examined the pharmacodynamics of new dosing regimens for polymyxin-B combination using a hollow-fiber infection model and determined that >4 mg/L polymyxin-B and 25 mg/L doripenem have synergistic antibacterial activities (Rao et al., 2016).

In the present study, we evaluated whether drug combinations consisting of amikacin/polymyxin-B plus sulbactam can reduce or close the MSW against MDR *A. baumannii* carrying OXA-23 genes. We showed that the combination of amikacin/polymyxin-B and the combination plus 4 mg/l sulbactam significantly reduced *f*T_MSW_ and increased *f*T_>MPC_. The combination of antibiotics with different antimicrobial mechanisms can achieve a better antibacterial effect. Multiple mechanisms of action in concert contributed to the reduction in MPC and MIC by amikacin/polymyxin-B/sulbactam. Polymyxin-B and sulbactam disrupt the stability of bacterial cell wall and cell membrane. The destruction of the stability and integrity of the bacterial outer membrane allows for more amikacin to enter the bacterial cells and inhibit protein synthesis (Han et al., 2019; Hussein et al., 2019; Zhao et al., 2021). The triple-antibiotic combination destabilizes bacterial cell structure and inhibits bacterial growth, thereby reducing MPC and MIC values, despite the presence of drug-resistant mutations.

This study shows that the utilization of antibiotic combination in the treatment of MDR *A. baumannii* infections is quite complex. Optimization of both the dose and route of administration should take into account the PD parameters associated with suppression of resistance. In some cases, drug combination may result in convergence of MPC and MIC but drug concentrations at the infection site may not be sufficient to even eradicate less resistant bacteria subpopulation. High-dose sulbactam regimens provide sufficient penetration into the lung tissues to achieve their target PD indices, whereas polymyxins and amikacin, due to their high molecular mass and hydrophilicity (Safdar, 2010; Zhu et al., 2021), have low ELF penetration after an IV administration. Low tissue drug concentrations in the lung after an IV administration will lead to treatment failure (Zhu et al., 2021). Aerosol delivery can improve drug concentration in the ELF and also reduce systemic toxicities (Cipolla and HKJPPA, 2013).

Amikacin/polymyxin-B and amikacin/polymyxin-B/meropenem combinations are used to treat bloodstream infections (BSIs); the combination of at least amikacin/polymyxin-B was recently shown in a retrospective study to be associated with survival benefit compared with monotherapy against *Klebsiella pneumoniae* carbapenemase-producing *K. pneumoniae* BSIs (Medeiros et al., 2019). No safety concern was reported in this study.

There are few randomised clinical trials addressing combination therapy: two studies investigated colistin with rifampin (Aydemir et al., 2013; Durante-Mangoni et al., 2013) and one examined meropenem with colistin (Paul et al., 2018) in carbapenem-resistant and extensively drug-resistant *A. baumannii* ventilator-associated pneumonia and severe infections; they showed no statistical significant clinical benefit. Our predictions of free drug concentrations in the blood and lungs after intravenous injections were consistent with these findings of no clinical benefits in lung infections due to extremely drug-resistant *A. baumannii* but the benefits of combination can be realized in BSIs. Caution is warranted when extrapolating the findings of *in vitro* studies to clinical benefits, given the difference in drug combination and pathogens.

This analysis has its limitations that often plague an MIC-based PK/PD index. The application of MPC and MSW results are based on threshold concentrations. In a dynamic system, bacteria response to antibiotics whether being killed or becoming resistant depends not only on concentrations being above or below a threshold but rather on the exposure profiles over time (Rayner et al., 2021). The threshold values also do not reflect dosing frequency or treatment duration. Another limitation of the present study is the lack of simulated drug concentrations in the ELF from inhaled amikacin and polymyxin-B. This limitation arose from the lack of population PK models developed for inhaled polymyxin and amikacin in humans that can predict ELF drug concentrations. The model for amikacin liposome inhalation suspension measures drug in sputum of patients in mg/g unit but was not translatable to concentrations in ELF that are often reported in mg/L unit (Rubino et al., 2021) whereas another model for nebulized amikacin only predicts drug concentration in the blood (Petitcollin et al., 2016). The model for aerosolized polymyxin-B was developed in a mouse infection model and is not relevant for humans (Lin et al., 2017).

The complexity of the lung structure and tissue microanatomy may affect the accuracy of our simulated drug concentrations in the ELF after intravenous administration. Our conclusion on drug exposures in the lung, however, is consistent with clinical observations of treatment failures in lung infections (Sweeney and Kalil, 2019). Drug administrations that can increase local antibiotic concentrations in the lung have attracted much interest recently (Wood and Swanson, 2017). A recent meta-analysis indicated advantage of nebulized amikacin as an adjunctive treatment of gram-negative pneumonia in mechanically ventilated patients without additional risk of nephrotoxicity (Qin et al., 2021). The international consensus guidelines for the optimal use of polymyxins recommended that inhaled polymyxins may be used adjunctively with intravenous polymyxins to treat hospital acquired pneumonia and ventilator-acquired pneumonia (Tsuji et al., 2019). The European guidelines for the management of HAP/VAP have recommendations for the use of aerosolized antibiotics in HAP/VAP (Rello et al., 2017); these are weak recommendations that are based primarily on observational studies and not randomized controlled trials.

This study provides a framework for pharmacodynamic evaluation of drug-resistant mutant suppression in an antimicrobial co-administration setting. The results thereby lay the groundwork for additional clinical evaluation.

## Data availability statement

The data presented in the study are deposited in the NCBI BioProject repository, accession number PRJNA868906.

## Author contributions

All authors contributed to the design of the study, acquisition, or analysis of data, drafted or revised the article for intellectual content, and approved the final version.

## Funding

This work was supported by a grant from Shandong Provincial Natural Science Foundation (ZR2019BC025).

## Conflict of interest

The authors declare that the research was conducted in the absence of any commercial or financial relationships that could be construed as a potential conflict of interest.

## Publisher’s note

All claims expressed in this article are solely those of the authors and do not necessarily represent those of their affiliated organizations, or those of the publisher, the editors and the reviewers. Any product that may be evaluated in this article, or claim that may be made by its manufacturer, is not guaranteed or endorsed by the publisher.

## Supplementary material

The Supplementary material for this article can be found online at: https://www.frontiersin.org/articles/10.3389/fmicb.2022.1013939/full#supplementary-material
