# Supplementary material for: Effects of amikacin, polymyxin-B, and sulbactam combination on the pharmacodynamic indices of mutant selection against multi-drug resistant Acinetobacter baumannii
Source: Front Microbiol. 2022 Oct 20;13:1013939. doi: 10.3389/fmicb.2022.1013939 (PMC9632654; doi:10.3389/fmicb.2022.1013939)
Supplement: Supplementary file 1 [file Data_Sheet_1.pdf]

Supplemental Materials for

Effects of amikacin, polymyxin and sulbactam combination on the pharmacodynamic indices of mutant selection against multi-drug resistant *Acinetobacter baumannii*

Shixing ZHU[1#], Chu SONG[1#], Jiayuan ZHANG[1], Shuo DIAO[1], M. Tobias HEINRICHS[2], Frederico S. MARTINS[3], Zhihua LV[1,4*], Yuanqi ZHU[5], Mingming YU[1,4*], Sherwin K. B. SY[6*]

1 School of Medicine and Pharmacy, Ocean University of China, Qingdao 266003, PR China

2 Department of Pharmaceutics, College of Pharmacy, University of Florida, Gainesville, Florida, USA

3 Faculty of Pharmaceutical Sciences of Ribeirão Preto, University of São Paulo, São Paulo, Brazil

4 Laboratory for Marine Drugs and Bioproducts of Qingdao National Laboratory for Marine Science and Technology, Qingdao 266003, PR China

5 Department of Laboratory Medicine, the Affiliated Hospital of Qingdao University, Qingdao 266003, PR China

6 Department of Statistics, State University of Maringá, Maringá, Paraná, Brazil

Description of population pharmacokinetic models

Table S1 lists the parameters of the population pharmacokinetic models for amikacin, polymyxin-B and sulbactam.

The population pharmacokinetic model for amikacin was a two-compartment model previously developed using data collected from critically ill patients with suspected ventilator-associated pneumonia (1). This model was used because both creatinine clearance (CL_{CR}) and body weight (WT) are covariates of the model parameters and amikacin is dosed by WT.

The model for polymyxin-B in critically ill patients was a 2-compartment model parameterized on weight-scaled parameters (2). Individual parameters were obtained by multiplying weight-scaled parameters with the virtual patient’s weight.

The model for sulbactam was a 2-compartment model based on that reported for community-acquired pneumonia patients (3). CL_{CR} and WT were covariates of clearance and peripheral volume, respectively.

Table S1: Summary population pharmacokinetic parameters of antibiotics used in the simulation

**Drug**	**Amikacin IV**	**Polymyxin-B IV**	**Sulbactam IV**
Reference	Burdet et al. 2014(1)	Sandri et al. 2013 (2)	Soto et al. 2014(3)
Population	60 critically ill patients with ventilator-associated pneumonia	24 critically ill Brazil patients	47 Japanese patients with community acquired pneumonia
CL_{CR} (mL/min)	82 (4-412)	33 (10–143)	71.0（34.6–176）
Age (yrs)	28-84	21-87	28-85
Infusion time (h)	0.5 h infusion	1 – 4 h	0.5 h infusion
Dosing regimen	20 mg/kg	0.45–3.38 mg/kg/day q12h or 24h	3g
No. compartment	2	2	2
Pop PK parameters	CL (L/h) = 4.3 × $(CLcr/82)^{0.7}$<br>CV% CL = 30<br>V_C (L) = 15.9 × $(WT/78)^{0.9}$ × $(PFratio/169)^{0.4}$<br>CV% V1 = 20<br>Q (L/h) = 12.1<br>CV% Q = 30<br>V_P (L) = 21.4<br>CV% V_P = 50	CL = 0.0276 L/h/kg<br>CV% CL = 32.4%<br>Q = 0.146 L/h/kg<br>CV% Q = 50.4%<br>V_C = 0.0939 L/kg<br>CV% V_C = 73.3%<br>V_P = 0.330 L/kg<br>CV% V_P = 70.1%	CL (L/h) = 10.4 × $(CLcr/71)^{0.701}$<br>CV% CL = 15.2<br>V_C (L) = 10.2<br>Q (L/h) = 4.58<br>V_P (L) = 4.04 (WT/51)<br>CV% V_P = 14.8
Covariates	CL_{CR}, WT	Not available	CL_{CR}, WT

CL_{CR}, creatinine clearance; WT, body weight; CV, coefficient of variation; CL, drug clearance; Q, intercompartmental clearance; V_C, central volume; V_P, peripheral volume.

Table S2: Resistance genes in the *A. baumannii* clinical isolates

**Resistance phenotype**	**Isolate A**	**Isolate C**	**Isolate E**	**Isolate F**	**Isolate G**	**Isolate 2**	**Isolate 12**	**Isolate 13**	**Isolate 20**	**Isolate 21**	**Isolate 22**
Ribosome protection	*msr (E)*	*msr (E)*	*msr (E)*	*msr (E)*	*msr (E)*	*msr (E)*	*msr (E)*	*msr (E)*	*msr (E)*	*msr (E)*	*msr (E)*
Efflux proteins	*tet (B)*	*tet (B)*	*tet (B)*	*tet (B)*	*tet (B)*	NA	*tet (B)*	*tet (B)*	*tet (B)*	*tet (B)*	NA
β-lactamase	*bla*OXA-66<br>*bla*TEM-1D<br>*bla*ADC-25<br>*bla*OXA-23	*bla*TEM-1D<br>*bla*ADC-25<br>*bla*OXA-66<br>*bla*OXA-23	*bla*OXA-23<br>*bla*ADC-25<br>*bla*TEM-1D<br>*bla*OXA-66	*bla*TEM-1D<br>*bla*OXA-66<br>*bla*ADC-25<br>*bla*OXA-23	*bla*OXA-66<br>*bla*ADC-25<br>*bla*TEM-1D<br>*bla*OXA-23	*bla*ADC-25<br>*bla*oXA-23<br>*bla*TEM-1D<br>*bla*OXA-66	*bla*OXA-66<br>*bla*OXA-23<br>*bla*TEM-1D<br>*bla*ADC-25	*bla*OXA-23<br>*bla*OXA-66<br>*bla*ADC-25<br>*bla*TEM-1D	*bla*OXA-23<br>*bla*OXA-66<br>*bla*ADC-25	*bla*ADC-25<br>*bla*OXA-66<br>*bla*OXA-23	*bla*OXA-23<br>*bla*TEM-1D<br>*bla*OXA-66<br>*bla*ADC-25
Sulphonamide resistance marker	*sul1*	NA	*sul2*	*sul2*	*sul2*	*sul1*	*sul1*<br>*sul2*	*sul1*	*sul1*	*sul2*	*sul1*
Aminoglycoside modifying enzyme	*aadA1*<br>*aac (6')-Ib-cr*<br>*aph (3")-Ib*<br>*aac (6')-Ib3*<br>*aph (6)-Id*<br>*aph (3')-Ia*<br>*aph (3")-b*	*aph (3")-Ib*<br>*aph (6)-Id*	*aph (3")-Ib*<br>*aph (3")-Ib*<br>*aph (3")-Ib*<br>*aph (3")-Ib*<br>*aph (6)-Id*	*aph (6)-Id*<br>*aph (3")-Ib*<br>*aph (3')-Ia*	*aph (3')-Ia*<br>*aph (6)-Id*<br>*aph (3")-Ib*	*aadA1*<br>*aac (3)-Ia*<br>*aph (3)-Ia*<br>*aac (6)-Ib-cr*<br>*aac (6)-Ib3*	*aph (3")-b*<br>*aph (3")-Ib*<br>*aph (3')-Ia*<br>*aph (6)-Id*<br>*aph (3")-b*	*aac (6)-Ib3*<br>*aac (6")-Ib-cr*<br>*aph (3")-Ib*<br>*aph (6)-Id*	*aph (3")-Ib*<br>*aadA1*<br>*aph (6)-Id*<br>*aph (3')-Ia*<br>*aph (3")-b*<br>*aac (6')-b-cr*<br>*aac (6")-Ib3*	*aph (3")-Ib*<br>*aph (3")-b*<br>*aph (6)-Id*	*aadA1*<br>*aac (6")-Ib3*<br>*aac (6")-b-cr*<br>*aac (3)-1*<br>*aph (3')-Ia*
Aminoglycoside resistance methylase	*armA*	*armA*	*armA*	*armA*	*armA*	*armA*	NA	*armA*	*armA*	*armA*	*armA*

Figure S1: Probability of steady-state amikacin C_{min} ≥ 10 mg/L. Amikacin dosing regimens by renal functions are listed in Table 1.

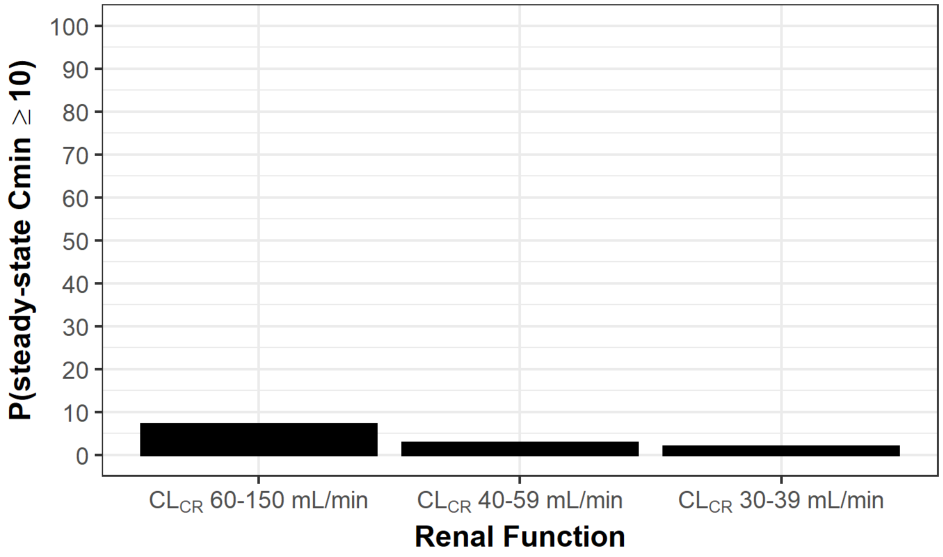

References

1. Burdet C, Pajot O, Couffignal C, Armand-Lefevre L, Foucrier A, Laouenan C, Wolff M, Massias L, Mentre F. Population pharmacokinetics of single-dose amikacin in critically ill patients with suspected ventilator-associated pneumonia. European journal of clinical pharmacology. 2015 Jan;71(1):75-83. doi:10.1007/s00228-014-1766-y. Cited in: Pubmed; PMID 25327505.

2. Sandri AM, Landersdorfer CB, Jacob J, Boniatti MM, Dalarosa MG, Falci DR, Behle TF, Saitovitch D, Wang J, Forrest A, Nation RL, Zavascki AP, Li J. Pharmacokinetics of polymyxin B in patients on continuous venovenous haemodialysis. The Journal of antimicrobial chemotherapy. 2013 Mar;68(3):674-7. doi:10.1093/jac/dks437. Cited in: Pubmed; PMID 23179561.

3. Soto E, Shoji S, Muto C, Tomono Y, Marshall S. Population pharmacokinetics of ampicillin and sulbactam in patients with community-acquired pneumonia: evaluation of the impact of renal impairment. British journal of clinical pharmacology. 2014 Mar;77(3):509-21. doi:10.1111/bcp.12232. Cited in: Pubmed; PMID 24102758.
